# Elevation of serum human epididymis protein 4 (HE4) and N-terminal pro-B-type natriuretic peptide (NT-proBNP) as predicting factors for the occurrence of acute kidney injury on chronic kidney disease: a single-center retrospective self-control study

**DOI:** 10.3389/fphar.2023.1269311

**Published:** 2023-09-11

**Authors:** Jinye Song, Ling Chen, Zheping Yuan, Xuezhong Gong

**Affiliations:** Department of Nephrology, Shanghai Municipal Hospital of Traditional Chinese Medicine, Shanghai University of Traditional Chinese Medicine, Shanghai, China

**Keywords:** human epididymis protein 4, N-terminal prohormone of B-type natriuretic peptide, acute kidney injury, chronic kidney disease, biomarkers

## Abstract

**Objectives:** To evaluate whether novel biomarkers of renal injury, serum HE4 and NT-proBNP could predict acute kidney injury (AKI) on chronic kidney disease (CKD) (A on C) and assess the specificity and efficiency of serum creatinine (SCr), HE4 and NT-proBNP in identifying potential AKI. Meanwhile, the potential early-warning value of HE4 and NT-proBNP in CKD patients was explored.

**Methods:** We performed a single-center, retrospective cohort study of 187 adult CKD patients. 32 AKI (grades 1–2) patients with pre-existing CKD (stages 3–5) were Group 1, 59 patients of CKD (stages 4–5) were Group 2. Another 96 patients of CKD (stages 1–3) were Group 3. All patients received general treatments, Group 1 patients received Chinese herb formulation (Chuan Huang Fang-Ⅱ, CHF-Ⅱ) simultaneously. These 155 CKD (stages 1–5) without AKI patients were observed for descriptive analysis.

**Results:** HE4 in Group 1 (860.63 ± 385.40) was higher than that in Group 2 (673.86 ± 283.58) before treatments. BUN, SCr, UA, NGAL, IL18, HE4 and NT-proBNP in Group 1 were lower, while eGFR was higher (*p* < 0.01, after vs. before treatments). In Group 1, both HE4 and NT-proBNP were positively correlated with SCr (respectively *r* = 0.549, 0.464) before treatments. The diagnostic performance of serum HE4 and NT-proBNP for A on C was 351.5 pmol/L, 274.5 pg/mL as the optimal cutoff value Area Under Curve (AUC) 0.860 (95% CI: 0.808 – 0.913, *p* < 0.001), [AUC 0.775 (95% CI: 0.697 – 0.853, *p* < 0.001), with a sensitivity and specificity of 100% and 66.5%, 87.5% and 48.8%, respectively]. In Group 2, serum HE4 was correlated with SCr (*r* = 0.682, *p* < 0.01) before treatments. Serum HE4 and NT-proBNP were elevated in advanced CKD stages, and were increased as CKD stages progressed with statistical significance.

**Conclusion:** This work indicated serum HE4 and NT-proBNP should elevate in A on C and CKD patients, HE4 is positively correlated with the disease severity, and patients with higher HE4 and NT-proBNP usually have poorer prognosis. Thus, serum HE4 and NT-proBNP are impactful predictors of A on C. Additionally, serum HE4 and NT-proBNP have the potential to evaluate clinical efficacy of A on C.

## 1 Introduction

Acute kidney injury (AKI) is a common syndrome characterized by a rapid decline in the glomerular filtration rate (GFR), which is associated with the development of chronic kidney disease (CKD) and poor prognosis. It has become one of the most debilitating complications and a public health problem threatening human health worldwide (Sato et al., 2020; Xie et al., 2020). Approximately 13.3 million AKI cases occur globally every year, leading to approximately 1.7 million deaths annually, and 85% of the patients belong to developing countries (Mehta et al., 2015; Liu et al., 2021). The morbidity rate of AKI is 3%–5% in the general inpatient population and 30%–50% in intensive care units. AKI has been accepted as an important public health problem worldwide. CKD is the common outcome of various kidney diseases and AKI progression, and the number of CKD patients is increasing globally. The prevalence of CKD in China is approximately 10.8% (Cao et al., 2023). The morbidity rate of CKD complicated with AKI accounts for 12.7%–35.5% of the total AKI cases (Prakash et al., 2015). Previously, CKD and AKI were believed to exist independently; however, increasing evidence shows that CKD and AKI are closely linked or have an influence on each other. AKI is an obvious risk factor for accelerating CKD progression to end-stage renal disease (ESRD), and early identification and intervention of A on C patients is crucial (He et al., 2017; Huang, 2018).

Currently, the clinical diagnosis of AKI is based on the serum creatinine (SCr) level and urine volume; however, these parameters are easily affected by the patient’s age, body weight, body fluid balance, and other factors. Therefore, early detection of renal injury is difficult. Additionally, patients with CKD have glomerular and renal tubule damage to a certain extent, and common indicators such as SCr and urine volume have limited value in the early prediction of AKI (Huang, 2018). Furthermore, the sensitivity of diagnosing AKI is poor, leading to missed diagnosis. Currently, there is a shortage of specific therapeutic drugs for AKI, and the conventional therapeutic principles of modern medicine focus on controlling the etiology, correcting the internal reversible factors, and alleviating the symptoms. AKI has attracted increasing attention due to its high incidence and poor prognosis. With the improvement of medical resources, researchers expect to find better biomarkers for the early prediction or detection of AKI.

N-terminal pro-B-type natriuretic peptide (NT-proBNP), a member of the diuretic natriuretic peptide family secreted by the heart, regulates the homeostasis of blood pressure and blood volume and has a diuretic effect. NT-proBNP correlates with the occurrence of AKI and may be of great value as a biomarker for the early prediction of AKI (Zhang et al., 2022). HE4, a novel tumor marker, has good predictive value for the progression of ovarian cancer (DeBoer et al., 2018). Recently, its expression was reported in the kidneys (Huang et al., 2020; Luo et al., 2020). The HE4 level in patients with renal impairment increases significantly, which could be related to the progression of CKD to some extent; however, the test results of HE4 could be influenced by renal function. HE4 indirectly inhibits the degradation of type I collagen (Nagy et al., 2016; Wang et al., 2019) and causes infiltration and polarization of M2 macrophages (Rowswell-Turner et al., 2021), which could promote the progression of renal fibrosis (Jiao et al., 2021a; Jiao et al., 2021b; An et al., 2023). Macrophages are the main immune cells in healthy kidneys and are considered key members in the pathogenesis of AKI (Li and Gong, 2023). HE4 expression is upregulated in renal tissues with fibrosis, and the serum HE4 level of the patients increases significantly (Wang et al., 2018), suggesting that HE4 may be a potential marker for early diagnosis and evaluation of CKD.

Traditional Chinese Medicine (TCM) treatment of AKI and CKD is historical in China. Rhubarb has shown particular efficacy in the clinical treatment of AKI and CKD. In the 1990s, studies showed that rhubarb delayed the progression of chronic renal failure, stabilized renal function, alleviated azotemia, and improved the patient’s nutritional status, symptoms, and physical strength (Zheng, 1985; Li, 1991). Our previous data indicated that a Chinese herbal formulation, Chuan Huang Fang (CHF) containing rhubarb, could exert a nephroprotective action in A on C patients (Gong et al., 2020; Gong et al., 2021; Chen et al., 2022). Furthermore, we conducted research on CHF against AKI in animal and cell models. CHF could effectively suppress the clinical dose of trivalent arsenic that causes renal toxicity, and its molecular mechanism was associated with the inhibition of caspase 3-induced renal tubular epithelial cell apoptosis (Gong et al., 2019; Chen and Gong, 2022a). We also investigated the mechanism of the Zhida Huang (prepared rhubarb)-Chuanxiong (Ligusticum wallichii) drug pair on tubular epithelial cell apoptosis in contrast-induced AKI (CI-AKI) rats (Gong et al., 2013). We found that activation of the p38MAPK pathway played an important role in the pathogenesis of CI-AKI, and the Zhida Huang-Chuanxiong drug pair might alleviate renal damage in CI-AKI rats by inhibiting the activation of the pathway. Based on this result, we explored the effect of the drug pair on the nuclear factor erythroid 2-related factor2/hemeoxygenase-1(Nrf2/HO-1) pathway (Gong et al., 2017). The Nrf2/HO-1 pathway was activated and involved in the process of AKI, and the Zhida Huang-Chuanxiong drug pair could activate the nephroprotective effects in the CI-AKI rats by inhibiting this pathway. Our recently published data (Li and Gong, 2023) on Rhizoma Chuanxiong and Radix et Rhizoma Rhei (rhubarb) against AKI and renal fibrosis based on network pharmacology and experimental validation shows that this herbal drug pair might inhibit tubular epithelial cell apoptosis and improve AKI and renal fibrosis by inhibiting p38 MAPK/p53 signaling.

Rhubarb plays an important role in the process of alleviating the progression of AKI and CKD, and it has been widely used in treating these conditions in China for many years. For instance, the widely used drugs in China, such as Shenkang injection and Shenshuaining capsules, include rhubarb. In order to further improve clinical efficacy, CHF was optimized to form CHF-Ⅱ, and current small-sample clinical efficacy observation was conducted to evaluate the actual effect of CHF-Ⅱ on A on C.

To the best of our knowledge, no study to date has investigated the utility of serum HE4 and NT-proBNP in predicting the incidence of A on C. Accordingly, we aimed to assess the possible predictive ability of serum HE4 and NT-proBNP levels to identify acute deterioration of renal function in CKD patients.

Therefore, this study intends to explore the potential early prediction value of serum HE4 and NT-proBNP in patients with CKD alone and those with A on C. Furthermore, we investigated their expression levels in different clinical stages of CKD and compared them with other renal function indicators.

## 2 Materials and methods

### 2.1 Patients

This single-center, retrospective cohort study included 187 adult CKD patients hospitalized in Shanghai Municipal Hospital of Traditional Chinese Medicine from June 2021 to December 2022. Patients with Grades 1–2 AKI with pre-existing Stages 3–5 CKD were categorized as Group 1 (*n* = 32), those with Stages 4–5 CKD were categorized as Group 2 (*n* = 59), and those with Stages 1–3 CKD were categorized as Group 3 (*n* = 96). All CKD patients underwent general treatment, and the Group 1 patients additionally received the Chinese herbal formulation CHF-Ⅱ.

The clinical data, including age, sex, medical history, blood pressure, and laboratory indices, were obtained by reviewing the medical records. This retrospective study was approved by the Ethics Committee of Shanghai Municipal Hospital of Traditional Chinese Medicine (No. 2020SHL-KYYS-60) and was conducted according to the Declaration of Helsinki principles.

### 2.2 Definition

#### 2.2.1 Diagnostic criteria for AKI

According to the “Kidney Disease: Improving Global Outcomes” (KDIGO) standards (KDIGO, 2013), Grade 1 AKI is defined by the following criteria: SCr level 1.5–1.9 times the baseline value or an increase of ≥0.3 mg/dL (26.5 μmol/L), or urine output (UO) < 0.5 mL/kg/h for 6–12 h.

Grade 2 AKI is defined by the following criteria: SCr level 2.0–2.9 times the baseline value, or UO < 0.5 mL/kg/h for ≥12 h.

Grade 3 AKI is defined by the following criteria: SCr level 3.0 times the baseline value or ≥4.0 mg/dL (353.6 μmol/L) or initiation of renal replacement therapy (RRT), or UO < 0.3 mL/kg/h for ≥24 h or anuria for ≥12 h. In patients aged <18 years, Grade 3 AKI is defined by estimated GFR (eGFR) < 35 mL/min/1.73 m^2^.

#### 2.2.2 Diagnostic criteria for CKD

CKD was defined as incident CKD during the observation period according to the following KDIGO guideline ^[16]^: “CKD is defined as abnormalities of kidney structure or function present for >3 months.”

CKD Stage 1 was defined as eGFR ≥90 mL/min/1.73 m^2^; CKD Stage 2 as eGFR 60–89 mL/min/1.73 m^2^; CKD Stage 3 as eGFR 30–59 mL/min/1.73 m^2^; CKD Stage 4 as eGFR 15–29 mL/min/1.73 m^2^; and CKD Stage 5 as eGFR <15 mL/min/1.73 m^2^. The CKD-Epidemiology Collaboration formula was used to calculate the eGFR of the CKD patients.

#### 2.2.3 Exclusion criteria

Patients with malignancy or acute cerebrovascular disease, those without laboratory measurements or medical records, and pregnant patients were excluded.

#### 2.2.4 Interventions

All patients were given a low-salt, high-quality, low-protein diet (protein intake, 0.6–0.8 g/kg/d). They all underwent general treatment to correct water, electrolyte, and acid-base balance disorders, control blood pressure, improve anemia, and correct renal bone diseases. Group 1 patients additionally received CHF-Ⅱ orally, twice a day for 2 weeks. Simultaneously, the concentrated CHF-Ⅱ solution was administered as an enema, once a day for 5 days, followed by rest for 2 days, and then 5 times a week. The pharmaceutical composition of CHF-Ⅱ mainly included prepared Zhida Huang, Chuanxiong, Codonopsis pilosula (Dangshen), Coptidis rhizome (Huanglian), and Smilacis glabrae (Tufuling), etc. The CHF-Ⅱ administered was produced by Jiangyin Tianjiang and Shanghai Wanshicheng Pharmaceutical Co., Ltd.; hence, the quantity and quality of medicine were guaranteed.

### 2.3 Methods

Peripheral venous blood specimens were collected in the early morning within 24 h after admission. For all patients, 10 mL of venous blood was collected and separated for 15 min at 3,500 rpm (14 cm from the core half diameter). The SCr, blood urea nitrogen (BUN), and uric acid (UA) levels were measured using Beckman Coulter chemistry analyzer AU5800 (Beckman Coulter Inc.). The serum HE4 and NT-proBNP levels were measured using electrochemiluminescence immunoassay, while urinary neutrophil gelatinase-associated lipocalin (NGAL) and interleukin (IL)-18 were measured using enzyme-linked immunosorbent assay. All the kits were purchased from Beijing Dingguo Changsheng Biotechnology Co., Ltd.

### 2.4 Statistical methods

All data were analyzed using Statistical Product and SPSS 22.0 statistical software. After data collection, the distribution of each parameter was examined to determine the appropriate statistical method for subsequent analyses. The NGAL and NT-proBNP values did not conform to normal distribution; therefore, they are presented as medians (interquartile ranges). Mann–Whitney *U* test was used to compare the differences between the groups. The measurement data are expressed as means ± standard deviations (SD). Spearman correlation analysis was used to evaluate the correlation of HE4 and NT-proBNP with SCr. The diagnostic value of each indicator for A on C was evaluated using the receiver operating characteristic (ROC) curve; *p*-values < 0.05 were considered statistically significant.

## 3 Results

### 3.1 Participant characteristics

The mean ± SD values of BUN, SCr, and UA did not differ significantly between Group 1 and Group 2. The average age of the participants in Group 1 and Group 2 was 62.1 ± 11.9 and 61.9 ± 11.6 years, respectively, and the number of male participants was 24 and 38, respectively. Poor medication adherence leading to aggravation of existing diseases was the main cause of AKI which accounted for 37.5% (Table 1), the main cause of CKD of both groups was chronic glomerulonephritis which accounted for 35.4% and 42.4% respectively, the causes of AKI and CKD of the two groups are shown in Table 1. The concomitant diseases in Groups 1 and 2 are presented in Table 2. No significant differences were observed in sex, age, and concomitant diseases between these two groups (*p* > 0.05). The baseline characteristics of the patients in Groups 1 and 2 were balanced, and the efficacy of this clinical study was comparable (Table 2).

**TABLE 1:** The causes of AKI and CKD of Group 1 and Group 2.

The causes of CKD of Group 1	Number (%)	The causes of AKI of Group 1	Number (%)	The causes of CKD of Group 2	Number (%)
chronic glomerulonephritis	11 (35.4%)	poor medication adherence leading to aggravation of existing diseases	12 (37.5%)	chronic glomerulonephritis	25 (42.4%)
IgA nephropathy	8 (25.0%)	infectious pneumonia	5 (15.6%)	IgA nephropathy	15 (25.4%)
diabetic nephropathy	7 (21.9%)	water-electrolyte imbalance	5 (15.6%)	diabetic nephropathy	12 (20.3%)
hypertensive nephropathy	4 (12.5%)	Diuretic, Contrast,NSAIDs-induced renal injury	4 (12.5%)	hypertensive nephropathy	5 (8.5%)
polycystic kidney disease	1 (3.2%)	heart failure	4 (12.5%)	polycystic kidney disease	2 (3.4%)
uric acid nephropathy	1 (3.2%)	acute gout attack	2 (6.3%)		

**TABLE 2:** Baseline characteristics of the participants.

Characteristics	Total (*n* = 91)	Group 1 (*n* = 32)	Group 2 (*n* = 59)
Male, n (%)	62 (68.1%)	24 (75.0%)	38 (64.4%)
Age (years), mean ± SD	62.3 ± 12.8	62.1 ± 11.9	61.9 ± 11.6
Concomitant diseases	85 (93.4%)	30 (93.8%)	55 (93.2%)
Hypertension, n (%)			
Type 2 diabetes mellitus, n (%)	51 (56.0%)	18 (56.3%)	33 (55.9%)

Group 1: Patients with Grades 1–2 AKI, and pre-existing Stages 3–5 CKD., Group 2: Patients with Stages 4–5 CKD. SD, standard deviation; AKI, acute kidney injury; CKD, chronic kidney disease.

### 3.2 Comparison of renal function indicators between Groups 1 and 2 before and after treatment

Before treatment, the mean ± SD values of BUN, SCr, and UA did not differ significantly between Group 1 and Group 2. The mean ± SD SCr values after treatment were 329.16 ± 113.61 μmol/L and 374.24 ± 148.47 μmol/L in Groups 1 and 2, respectively. Compared to the SCr level before treatment in Group 1, a significant decline was observed after treatment (*p* < 0.01); however, Group 2 showed no significant difference in the SCr levels before and after treatment (*p* < 0.05).

**FIGURE 1:**
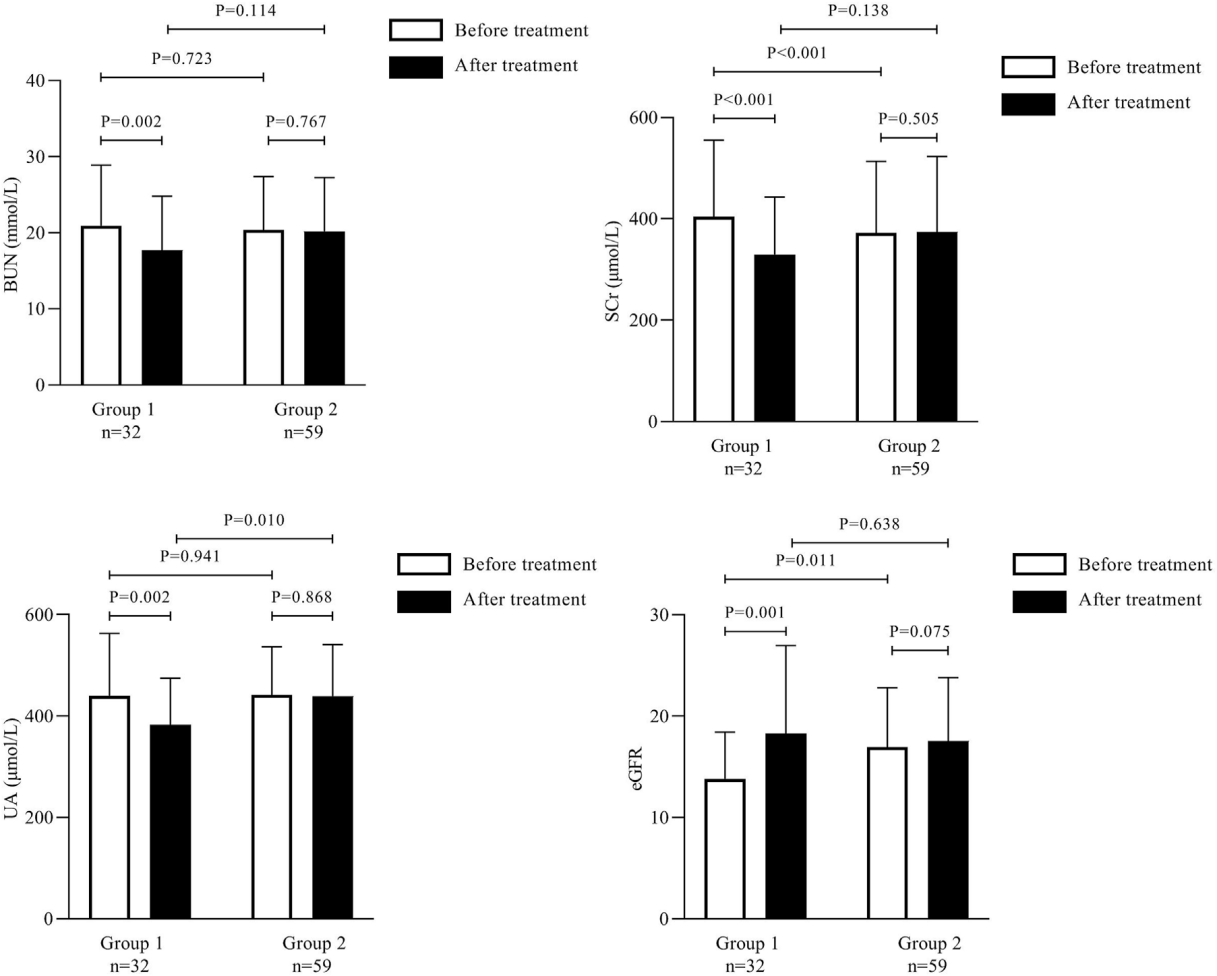
Comparison of the renal function indicators between Group 1 and Group 2 before and after treatment.

The mean ± SD values of BUN after treatment were 17.69 ± 7.14 mmol/L and 20.17 ± 7.06 mmol/L in Groups 1 and 2, respectively. The mean BUN value in Group 1 was significantly lower after treatment than that before treatment (*p* < 0.01). Meanwhile, the mean UA level before treatment in Group 1 was significantly lower than that after treatment (*p* < 0.01). Similarly, the eGFR improved significantly after treatment in Group 1 (*p* < 0.01). However, in Group 2, no significant differences were observed in the BUN, UA, and eGFR values before and after treatment (*p* < 0.05) (Table 3; Figure 1).

**TABLE 3:** Comparison of the renal function indicators between Group 1 and Group 2 before and after treatment.

Variables	Group 1 (*n* = 32)		Group 2 (*n* = 59)	
	Before treatment	After treatment	Before treatment	After treatment
SCr (µmol/L)	404.00 ± 151.18	329.16 ± 113.61**	371.59 ± 141.51	374.24 ± 148.47
BUN (mmol/L)	20.92 ± 7.98	17.69 ± 7.14**	20.34. ± 7.04	20.17 ± 7.06
UA (µmol/L)	440.13 ± 122.69	382.56 ± 92.00**	441.84 ± 94.38	439.34 ± 101.29^##^
eGFR (mL/min/1.73m^2^)	13.82 ± 4.62	18.30 ± 8.67**	16.93 ± 5.89	17.56 ± 6.26

Group 1: Patients with Grades 1–2 AKI, and pre-existing Stages 3–5 CKD., Group 2: Patients with Stages 4–5 CKD. SCr, serum creatinine; BUN, blood urea nitrogen; UA, uric acid; eGFR, estimated glomerular filtration rate. **Statistically significant difference from before treatment; *p* < 0.01 considered statistically significant. ##Statistically significant difference from Group 1; *p* < 0.01 considered statistically significant.

No significant differences were observed in the SCr, BUN, and eGFR values between Groups 1 and 2 after treatment (*p* > 0.05).

### 3.3 Various expressions of urine NGAL and IL-18 between Groups 1 and 2

To investigate the prediction performance of the existing AKI biomarkers for A on C, we evaluated the expression of NGAL and IL-18. Both these biomarkers were significantly elevated in Groups 1 and 2. Before treatment, the NGAL and IL-18 levels were significantly elevated in Group 1 when compared with Group 2 (*p* < 0.01).

The median NGAL level was significantly higher in Group 1 than in Group 2 (265.200 ng/mL [138.20–539.50] vs. 151.800 ng/mL [95.00–266.90]; *p* < 0.001); this level decreased significantly after treatment in Group 1 (203.950 ng/mL [113.50–487.70]; *p* < 0.01).

**FIGURE 2:**
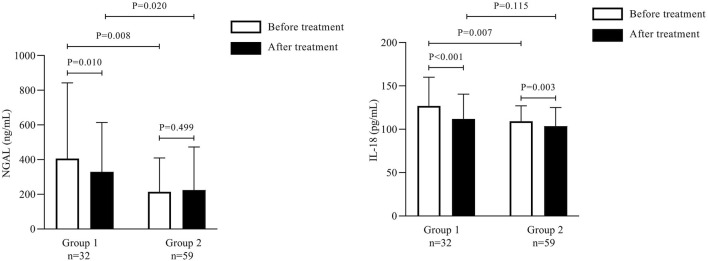
Comparison of urine neutrophil gelatinase-associated lipocalin and interleukin-18 between Group 1 and Group 2 before and after treatment.

In contrast, the median NGAL level in Group 2 did not differ significantly before and after treatment (Table 4). The IL-18 levels reduced significantly after treatment in Group 1 (112.06 ± 28.45 pg/mL) and Group 2 (103.63 ± 21.50 pg/mL) as compared to the levels before treatment (127.06 ± 33.01 pg/mL and 109.41 ± 17.65 pg/mL, respectively; *p* < 0.001) (Figure 2).

**TABLE 4:** Comparison of neutrophil gelatinase-associated lipocalin, interleukin-18, human epididymis secretory protein 4, and N-terminal pro-B-type natriuretic peptide between Group 1 and Group 2 before and after treatment.

Variables	Group 1 (n = 32)		Group 2 (n = 59)	
	Before treatment	After treatment	Before treatment	After treatment
NGAL (ng/mL), (P25, P75)	265.200 (138.2–539.5)	203.950 (113.5–487.7)**	151.800 (95.0–266.90)^##^	136.000 (93.0–287.7)
IL-18 (pg/mL), mean ± SD	127.06 ± 33.01	112.06 ± 28.45**	109.41 ± 17.65^##^	103.63 ± 21.50
HE4 (pmol/mL), mean ± SD	860.63 ± 385.40	737.59 ± 331.49*	673.86 ± 283.58^##^	684.64 ± 310.11
NT-proBNP (pg/mL), (P25, P75)	613.500 (293.3–3747.0)	435.000 (278.5–1693.5)**	529.000 (266.5–1225.5)	535.000 (331.0–1103.0)

Group 1: Patients with Grades 1-2 AKI, and pre-existing Stages 3–5 CKD., Group 2: Patients with Stages 4–5 CKD. NGAL, neutrophil gelatinase-associated lipocalin; IL, interleukin; HE4, human epididymis secretory protein 4; NT-proBNP, N-terminal pro-B-type natriuretic peptide; SD, standard deviation. *Statistically significant difference from before treatment; *p* = 0.01. **Statistically significant difference from before treatment; *p* < 0.01. ##Statistically significant difference between Group 1 and Group 2; *p* < 0.01.

### 3.4 Various expressions of HE4 and NT-proBNP between Groups 1 and 2

The mean serum HE4 level before treatment was significantly higher in Group 1 than in Group 2 (860.63 ± 385.40 pmol/mL vs. 673.86 ± 283.58 pmol/mL; *p* = 0.01); this level decreased significantly after treatment in Group 1 (737.59 ± 331.49 pmol/mL; *p* < 0.001).

**FIGURE 3:**
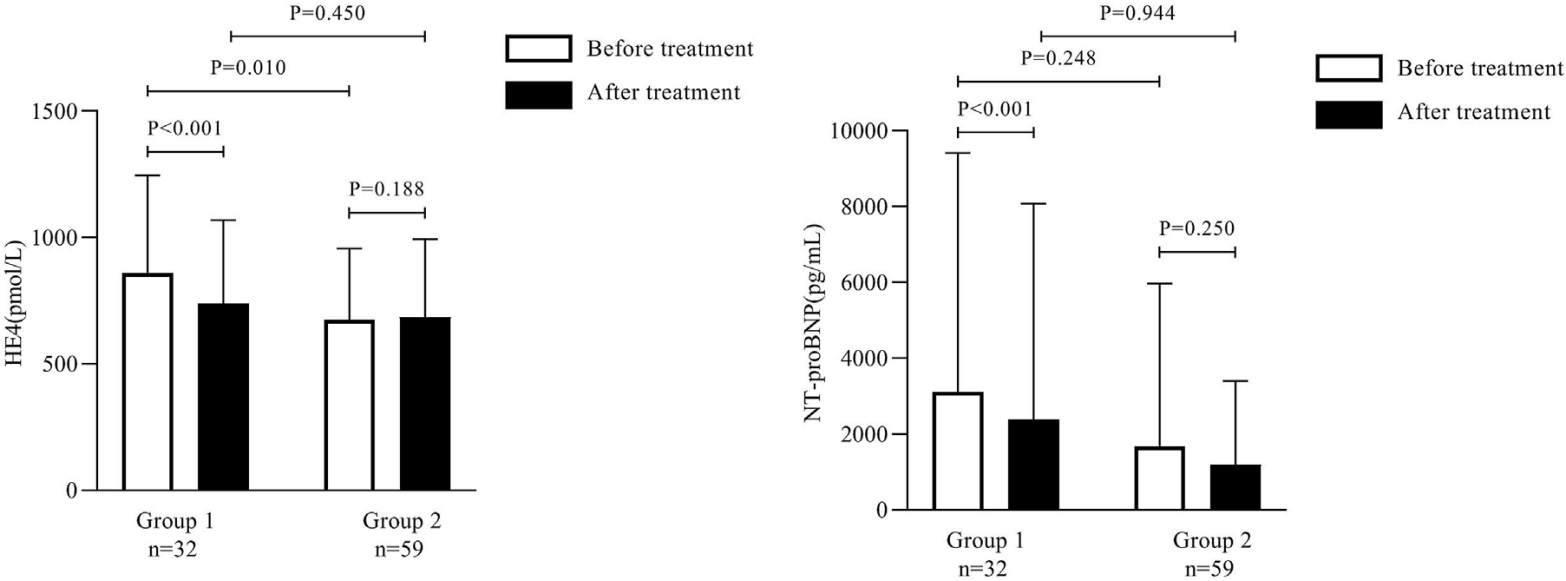
Comparison of human epididymis secretory protein 4 and N-terminal pro-B-type natriuretic peptide between Group 1 and Group 2 before and after treatment.

In contrast, the mean HE4 level in Group 2 did not differ significantly before and after treatment (Table 4; Figure 3). The median NT-proBNP level was significantly higher in Group 1 than in Group 2 before treatment (613.500 pg/mL [293.3–3747.0] vs. 529.000 pg/mL [266.5–1225.5]; *p* < 0.001); this level decreased significantly after treatment in Group 1 (435.000 pg/mL [278.5–1693.5]; *p* = 0.01).

In contrast, the median NT-proBNP level in Group 2 did not differ significantly before and after treatment (Table 4; Figure 3).

**FIGURE 4:**
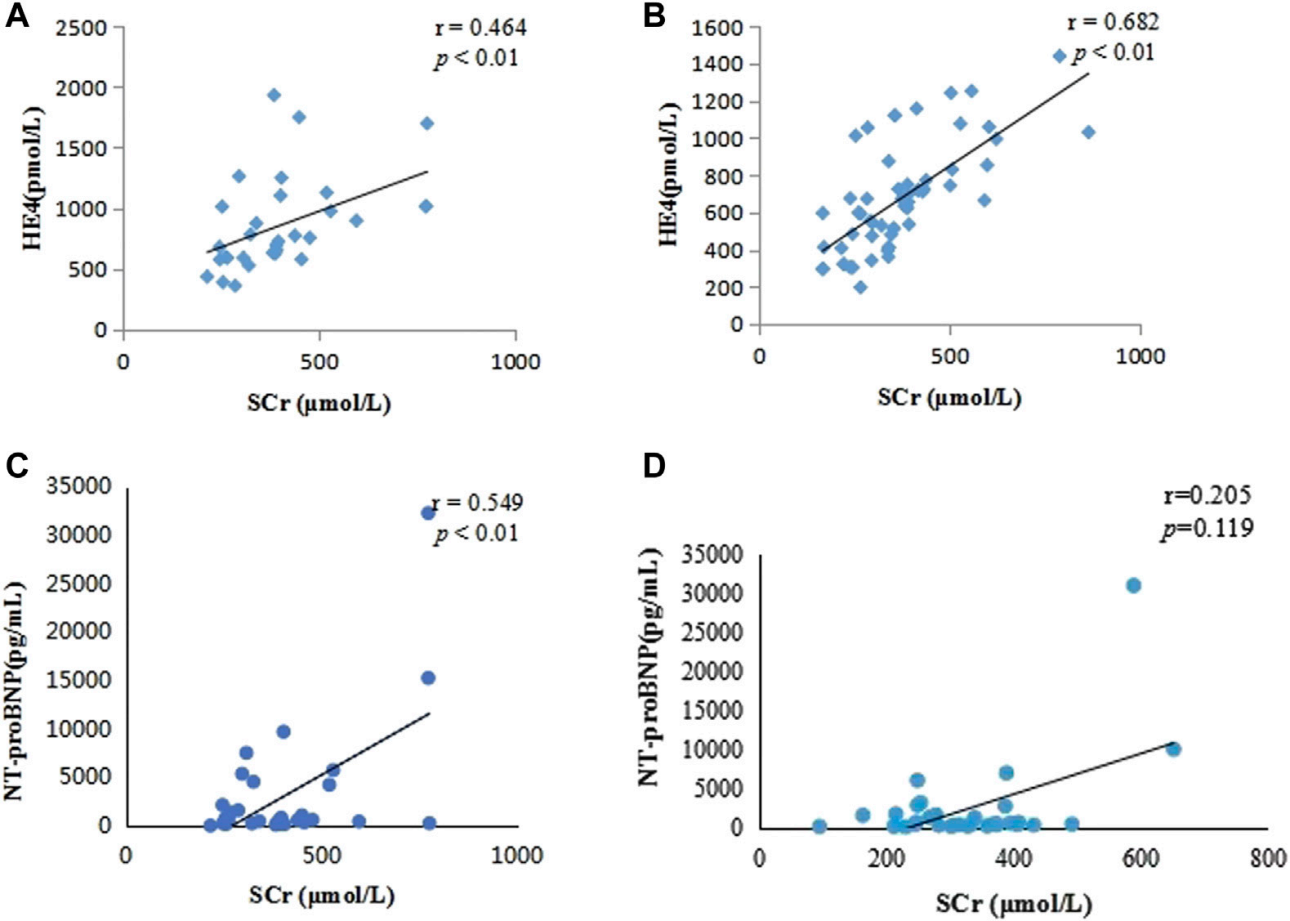
Scatterplots showing the correlation between human epididymis secretory protein 4 and serum creatinine in Group 1 **(A)** and Group 2 **(B)** and between N-terminal pro-B-type natriuretic peptide and serum creatinine in Group 1 **(C)** and Group 2 **(D)**.

Spearman correlation analysis revealed that serum HE4 correlated positively with SCr in Groups 1 and 2 before treatment (*r* = 0.464 and 0.682, respectively; *p* < 0.01). Moreover, NT-proBNP correlated positively with SCr (*r* = 0.549; *p* < 0.01) in Group 1 before treatment but not in Group 2 (*r* = 0.205; *p* = 0.119) (Figure 4).

**FIGURE 5:**
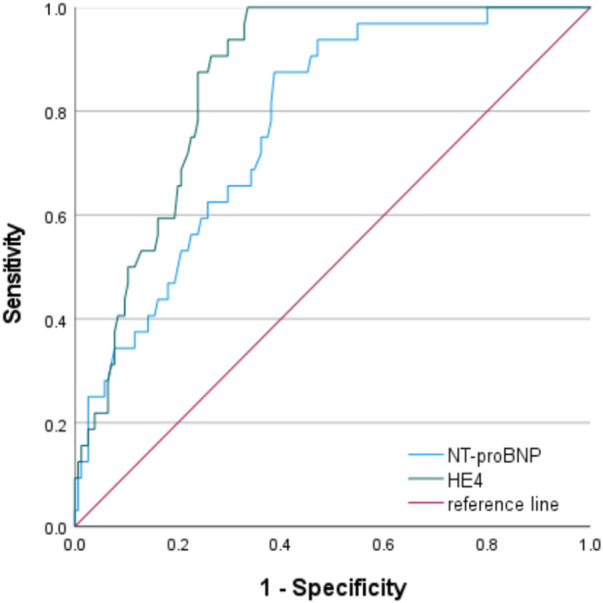
Receiver operating characteristic curves of human epididymis secretory protein 4 and N-terminal pro-B-type natriuretic peptide for the diagnosis of acute kidney injury on chronic kidney disease The AUC values of serum HE4 and NT-proBNP for A on C were 0.860 (95% CI: 0.808–0.913; *p* < 0.001) and 0.775 (95% CI: 0.697–0.853; *p* < 0.001), respectively, and their optimum cutoff values to predict A on C were 351.5 pmol/L and 274.5 pg/mL, respectively. (AUC, area under the curve; CI, confidence interval; HE4, human epididymis secretory protein 4; NT-proBNP, N-terminal pro-B-type natriuretic peptide; A on C, acute kidney injury on chronic kidney disease).

The ROC curve for diagnostic performance showed that the optimal cutoff value of serum HE4 for A on C was 351.5 pmol/L (area under the curve [AUC], 0.860; 95% confidence interval [CI]: 0.808–0.913; *p* < 0.001), with a sensitivity and specificity of 100% and 66.5%, respectively. The optimal cutoff value of serum NT-proBNP for A on C was 274.5 pg/mL (AUC, 0.775; 95% CI: 0.697–0.853; *p* < 0.001), with a sensitivity and specificity of 87.5% and 48.8%, respectively (Figure 5).

### 3.5 Comparison of renal function indicators and biomarkers in CKD patients

**FIGURE 6:**
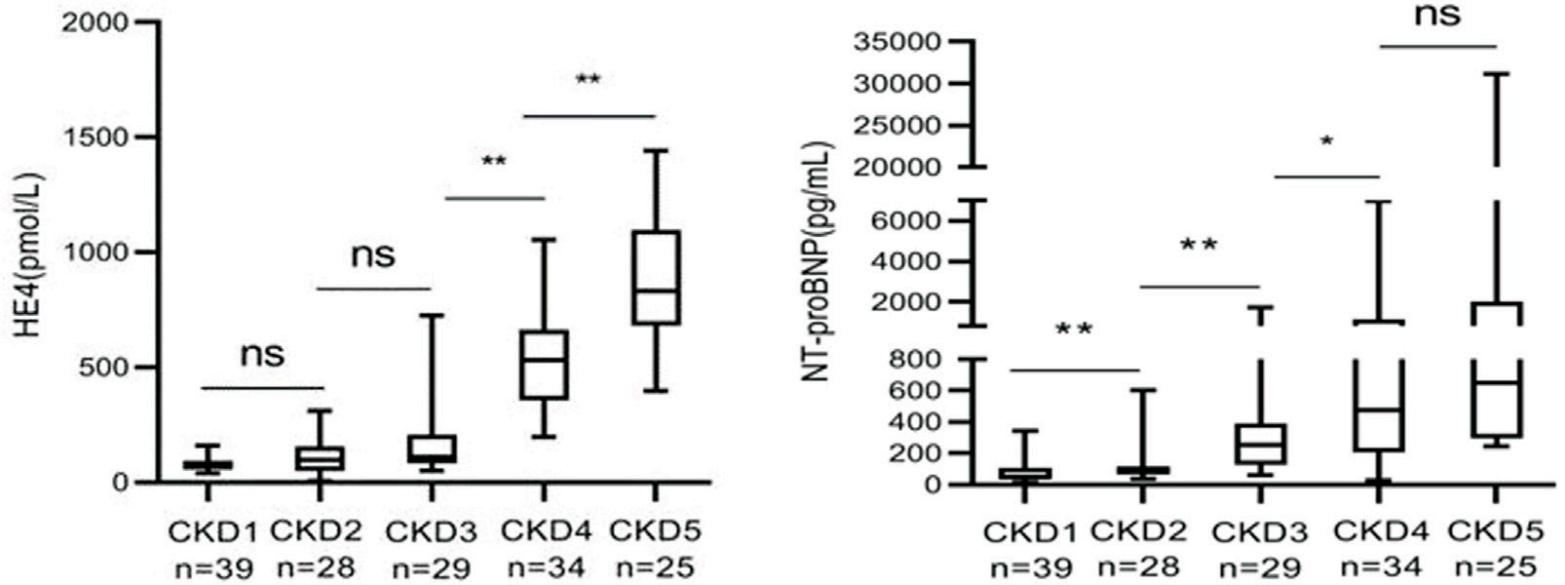
Comparison of human epididymis secretory protein 4 and N-terminal pro-B-type natriuretic peptide in chronic kidney disease patients (non-significant: *p* > 0.05; *p < 0.05; **p < 0.01).

We further categorized the 155 CKD patients (Stage 1, *n* = 39; Stage 2, *n* = 28; Stage 3, *n* = 29; Stage 4, *n* = 34; and Stage 5, *n* = 25) according to their eGFR values. The serum HE4 levels increased as the CKD stages progressed, with a statistically significant difference. Serum HE4 levels were obviously elevated in advanced CKD stages, suggesting serum HE4 as a novel biomarker for predicting the severity of CKD. However, no significant difference was observed in the HE4 levels between Stage 1 and Stage 2 CKD (*p* = 0.222) and between Stage 2 and Stage 3 CKD (*p* = 0.112) (Table 5; Figure 6).

**TABLE 5:** Clinical characteristics and laboratory tests of Group 2 and Group 3.

Variables	Total (*n* = 155)	CKD1 (*n* = 39)	CKD2 (*n* = 28)	CKD3 (*n* = 29)	CKD4 (*n* = 34)	CKD5 (*n* = 25)
Age (years)	63.11 ± 14.24	59.36 ± 12.90	66.71 ± 13.03	64.84 ± 15.19	61.35 ± 15.89	64.64 ± 12.24
Sex (male/female); n	155 (87/68)	39 (18/21)	28 (14/14)	29 (15/14)	34 (25/9)	25 (15/10)
Primary diseases						
Diabetes	77 (49.68%)	11 (28.21%)	10 (35.71%)	11 (37.93%)	20 (58.82%)	15 (60%)
Hypertension	106 (68.39%)	19 (48.72%)	19 (67.86%)	21 (72.41%)	27 (79.41%)	20 (80%)
Laboratory tests						
SCr (µmol/L)	204.59 ± 131.33	61.67 ± 12.76	88.11 ± 18.36^aa^	169.52 ± 41.37^bb^	299.09 ± 79.23^cc^	470.20 ± 148.94^d^
BUN (mmol/L)	12.76 ± 6.62	5.71 ± 1.81	6.98 ± 1.40^aa^	12.42 ± 3.70^bb^	17.83 ± 6.65^cc^	23.76 ± 6.16^d^
UA (µmol/L)	410.62 ± 77.99	332.68 ± 76.04	406.64 ± 77.41^aa^	455.78 ± 86.49^b^	452.09 ± 96.24	427.88 ± 91.87
eGFR (mL/min/1.73 m^2^)	59.18 ± 38.60	127.70 ± 25.40	70.59 ± 9.95^aa^	41.98 ± 9.34^bb^	20.79 ± 4.51^cc^	11.68 ± 2.46^d^
IL-18 (pg/mL)	212.86 ± 22.24	91.03 ± 18.37	102.07 ± 16.57^a^	99.41 ± 17.39	104.00 ± 16.08	152.76 ± 65.17^d^
NGAL (ng/mL)	132.20 (78.20–238.00)	62.50 (38.4–114.00)	115.50 (83.3–173.0)^aa^	250.00 (175.5–358.5)^bb^	123.00 (82.4–159.6)^cc^	226.00 (165.8–391.6)^d^
HE4 (pmol/L)	323.65 ± 274.65	77.34 ± 22.17	109.18 ± 61.03	149.49 ± 77.02	531.09 ± 151.38^cc^	868.04 ± 277.89^d^
NT-proBNP (pg/mL)	175.00 (75.00–510.00)	45.40 (36.2–110.0)	79.20 (64.3–117.8)^aa^	254.00 (126.5–390.5)^bb^	477.00 (208.3–1120.5)^c^	649.00 (295.0–2017.0)

Data are presented as n (%) or means ± standard deviations for normally distributed continuous variables and as medians (interquartile ranges) for non-normally distributed continuous variables. Group 2: Patients with Stages 4–5 CKD., Group 3: Patients with Stages 1–3 CKD. CKD1 (CKD, Stage 1), patients with eGFR ≥90 mL/min/1.73 m^2^; CKD2 (CKD, Stage 2), patients with eGFR, 60–89 mL/min/1.73 m^2^; CKD3 (CKD, Stage 3), patients with eGFR, 30–59 mL/min/1.73 m^2^; CKD4 (CKD, Stage 4), patients with eGFR, 15–29 mL/min/1.73 m^2^; CKD5 (CKD, Stage 5), eGFR <15 mL/min/1.73 m^2^. CKD, chronic kidney disease; SCr, serum creatinine; BUN, blood urea nitrogen; UA, uric acid; eGFR, estimated glomerular filtration rate; NGAL, neutrophil gelatinase-associated lipocalin; IL, interleukin; HE4, human epididymis secretory protein 4; NT-proBNP, N-terminal pro-B-type natriuretic peptide. ^a^ *p* < 0.05. ^aa^ *p* < 0.01, CKD1 vs. CKD2. ^b^ *p* < 0.05. ^bb^ *p* < 0.01, CKD2 vs. CKD3. ^c^ *p* < 0.05. ^cc^ *p* < 0.01, CKD3 vs. CKD4. ^d^ *p* < 0.01, CKD4 vs. CKD5.

Furthermore, the NT-proBNP levels increased as the CKD stages progressed, with a statistically significant difference. The serum NT-proBNP levels were obviously elevated in advanced CKD stages; however, no significant difference was observed in the serum NT-proBNP levels between Stage 4 and Stage 5 CKD (*p* = 0.069) (Table 5; Figure 6).

## 4 Discussion

To the best of our knowledge, this is the first study investigating the clinical significance of serum HE4 and NT-proBNP levels in A on C patients. This study found that serum HE4 and NT-proBNP levels were elevated in CKD patients, especially in A on C patients. Furthermore, SCr was positively associated with elevated serum HE4 and NT-proBNP levels in A on C patients. Moreover, serum HE4 and NT-proBNP were strongly associated with an increased risk of A on C and could be used as novel biomarkers for A on C diagnosis.

This study showed that CHF-Ⅱ as part of the integrated TCM and Western medicine has positive clinical efficacy in treating A on C, corroborating our previous findings (Chen and Gong, 2022a; Chen and Gong, 2022b; Chen et al., 2022). This study further confirms the clinical value of CHF-Ⅱ and is worthy of further research and promotion of its application. Furthermore, it shows that serum HE4 and NT-proBNP could help evaluate the effectiveness of the drug treatment regimens.

Several studies (Chen et al., 2017; Luo et al., 2018) reported that increased serum HE4 level is associated with decreased renal function in CKD patients. Using the AUC analysis, this study showed that serum HE4 and NT-proBNP are good indicators and predictors of AKI in CKD patients.

AKI and CKD are closely related. Although old age, nephrotoxic drugs, sepsis, chronic heart disease, and diabetes are common risk factors for AKI in clinical practice (Hsu et al., 2016; Liu et al., 2018), the most important risk factor is pre-existing CKD. The incidence of AKI in patients with CKD is nearly 10 times that of patients without CKD (Moo Park, 2014; Wonnacott et al., 2014). AKI can lead to new incidence of CKD, progression of existing CKD, increased risk of long-term morbidity, and increased mortality rate in patients with ESRD. AKI and CKD are risk factors for each other (Chawla et al., 2014); hence, it is crucial to improve the comprehensive understanding of A on C, perform early detection and intervention, and promote recovery of renal function. It is of great clinical significance to strengthen the early detection and diagnosis of A on C.

Privratsky et al. (2022) reported that the risk of postoperative AKI and its severity correlated positively with the risk of developing CKD or ESRD within 1 year after surgery, and preoperative CKD was an independent risk factor for postoperative AKI; however, there was no correlation with the severity of AKI. Hatakeyama et al. (2017) reported a positive correlation between AKI and glomerular dysfunction. Additionally, the frequency of AKI in patients with CKD is significantly higher than that in patients without CKD, especially after reaching Stage 3b of CKD (Zhang et al., 2019). Therefore, AKI increases the risk of developing CKD and may accelerate the progression of CKD to ESRD. Conversely, CKD itself is one of the main risk factors inducing AKI. Thus, AKI and CKD are interrelated and influence each other. CKD increases susceptibility to AKI, and the prognosis of CKD complicated with AKI is often poor.

The prevention and treatment principle of A on C focuses on reducing the incidence of AKI as much as possible, improving renal function to the greatest extent in the short term, correcting the influence of AKI on CKD, protecting renal function in the long term, and improving the survival rate. In CKD patients who have developed AKI, the cause of AKI should be corrected as soon as possible, and RRT should be performed at the earliest in severe cases. The incidence of A on C has been increasing every year. The interaction between CKD and AKI shows that A on C is a complex clinical syndrome.

The diagnostic means of modern medicine and TCM treatment have their advantages; hence, with the deepening of modern medical research, we must continue to look for sensitive diagnostic markers. Moreover, the continuous research on the curative effects of TCM in treating this disease as well as disease differentiation by Western medicine combined with syndrome differentiation by TCM can maximize the advantages of these two approaches and minimize the recovery time of renal injury, which is the key to treating this disease.

NT-proBNP, which is mainly derived from the ventricle, promotes sodium excretion and urination and has a strong vasodilatory effect, which can counteract the vasodilatory effect of the renin-angiotensin-aldosterone system. Cardiac dysfunction can greatly activate the natriuretic peptide system, and increased ventricular load leads to NT-proBNP release (Wang and Xu, 2020). When AKI occurs, the renal structure is damaged, NT-proBNP receptors are destroyed, and the ability to bind NT-proBNP is decreased, resulting in increased plasma-free NT-proBNP levels. The change in NT-proBNP levels is closely associated with renal function deterioration and prognosis (Wettersten et al., 2019).

NT-proBNP has been extensively studied in patients with cardiovascular disease. It was found that this indicator can predict the progression of AKI in patients with ST-segment elevation myocardial infarction or heart failure (Palazzuoli et al., 2014). In patients with cardiac arrest, NT-proBNP is considered a marker of cardiac and renal load and a risk factor for AKI after cardiac surgery. A prospective trial of ICU patients with non-cardiac causes concluded that NT-proBNP levels could predict the development of AKI (de Cal et al., 2011). Chou et al. (2015) measured the NT-proBNP levels 24 h after admission in 163 critically ill patients and showed that changes in NT-proBNP on the day of admission and after 24 h predicted the development of AKI; however, it did not adjust for disease severity and potential cardiac risk factors. Papanikolaou et al. (2014) measured the NT-proBNP concentrations in patients with sepsis and showed that the levels increased during sepsis and septic shock, which was attributed to the release of pro-inflammatory cytokines and biventricular dysfunction. The results showed that the NT-proBNP concentrations correlated independently with the AKI stage and RRT.

In AKI patients, the GFR and urine volume decrease, resulting in excessive volume load, which leads to increased NT-proBNP secretion by the ventricular myocytes. Recently, studies have further verified the threshold value of NT-proBNP for excessive volume load in AKI patients. It was reported that NT-proBNP in patients with AKI had a good correlation with the net ultrafiltration, and NT-proBNP levels were significantly lower in patients with hypotension after continuous RRT than in those without AKI (Cui et al., 2013). Yang et al. (2016) conducted a prospective study to detect serum HE4 levels in CKD patients. The relationship between HE4 and CKD progression was analyzed. Finally, it was confirmed that high expression of serum HE4 is associated with poor renal prognosis in CKD patients, suggesting that HE4 may be a serological marker for CKD progression. Wan et al. (2016) reported that the serum HE4 level in CKD patients was significantly higher than that in healthy individuals. The elevated HE4 level correlated closely with the CKD stage; the higher the HE4 level, the more severe the renal fibrosis. Correlation analysis in a previous study showed that HE4 correlated significantly with the degree of renal fibrosis (r = 0.938; *p* < 0.0001); the AUC was 0.99, which was higher than that of SCr (0.89). He et al. (2016) showed that the efficacy of serum HE4 in the early diagnosis of renal injury in male patients with CKD was higher than that of SCr and comparable to that of cystatin-C, which is a sensitive indicator reflecting the eGFR. Based on the results of the existing biological studies, we concluded that serum HE4 level increases significantly in CKD patients with renal fibrosis and correlates with the CKD stage (Lv et al., 2015).

The elevated level of HE4 in CKD patients is attributed to two factors. One is that as a small molecule, HE4 is filtered by the glomerulus, and when eGFR declines, the HE4 level rises inevitably. The other is that HE4 expression level in CKD patients increases significantly (Qu et al., 2016). This provides a new approach for the evaluation of chronic renal function injury.

The HE4 expression is upregulated in patients with renal tissue fibrosis, and the injection of HE4 neutralizing antibody in the mouse renal disease model can inhibit renal fibrosis and delay the progression of CKD in the mice (LeBleu et al., 2013; Wan et al., 2016). Studies have shown that when fibrosis changes occur in the kidneys of CKD patients, the intrinsic cells of the kidney will proliferate and differentiate to form myoblasts (Bai et al., 2019). The HE4 gene in myoblasts is significantly upregulated, resulting in increased HE4 protein secretion. Elevated HE4 inhibits the degradation of collagen I by inhibiting the activity of serine proteases (Prss23 and Prss35) and matrix metalloproteinases (MMP-2 and MMP-9) and accelerates the deposition of type I collagen in the kidneys, leading to renal fibrosis. There are two possible reasons for the increase in HE4 levels in CKD patients. First, the filtration and reabsorption of small molecule substances are significantly affected when the renal function is impaired, and the glomeruli cannot effectively clear HE4, thus the serum HE4 expression level increases significantly (Li et al., 2022). Second, when the kidney changes structurally, the formation of myofibroblasts and secretion of HE4 increases; this increased HE4 expression increases the accumulation of collagen in the extracellular matrix, which promotes the occurrence and development of renal fibrosis (Qu et al., 2016).

## 5 Conclusion

We conducted a retrospective study to investigate the levels of HE4 and NT-proBNP in A on C and CKD patients and of renal function parameters. We also compared the HE4 and NT-proBNP levels in CKD patients according to their clinical stages. Moreover, we analyzed the correlations of HE4 and NT-proBNP with the renal function parameters in A on C and CKD patients. Our study adds additional evidence that serum HE4 and NT-proBNP could be sensitive and specific biomarkers for diagnosing A on C.

This study extended its findings by revealing significant associations between elevated serum HE4 and NT-proBNP levels and the loss of renal function and decreased eGFR in A on C patients. Therefore, these findings suggest that serum HE4 and NT-proBNP levels are elevated as CKD progresses and that they could be valuable biomarkers in patients with a risk of CKD progression. Moreover, HE4 and NT-proBNP may be potential novel biomarkers for assessing treatment efficacy and effectiveness in A on C patients. The HE4 and NT-proBNP levels can help to identify the severity of A on C or CKD and are powerful predictors of rapid CKD progression.

Our study has certain limitations. First, it was a single-center study with a modest sample size. Second, no information was provided regarding the cause of CKD in the patients. The small number of patients with CKD might be insufficient to determine the reliability and generalizability of serum HE4 and NT-proBNP as biomarkers of A on C. Finally, although this study aimed to identify the potential biomarkers for predicting A on C, the underlying mechanism of these predictors in A on C remains unclear, which requires further investigation. Further investigations with larger samples are warranted to confirm these findings.

## Data Availability

The original contributions presented in the study are included in the article/Supplementary Material, further inquiries can be directed to the corresponding author.
